# Paediatric antiretroviral overdose: A case report from a resource-poor area

**DOI:** 10.4102/sajhivmed.v21i1.1094

**Published:** 2020-07-21

**Authors:** Bryan A. Ogoti, Angela A. Otedo, Thomas M. Chokwe

**Affiliations:** 1 Department of Anaesthesia, Faculty of Medicine, University of Nairobi, Nairobi, Kenya; 2 Department of Medical Services, Avenue Healthcare, Nairobi, Kenya

**Keywords:** antiretroviral, abacavir, lamivudine, poisoning, overdose, paediatrics, resource-poor

## Abstract

**Introduction:**

Toxic side effects from antiretroviral overdose in children have not been widely reported. Antiretroviral drugs are widely used as oral medications throughout sub-Saharan Africa.

**Patient presentation:**

We describe the clinical presentation and management of a 3-year-old male in rural Kenya, who accidentally overdosed on abacavir/lamivudine combination pills. The number of pills taken was approximately 250 tablets, that is 15 g of abacavir and 7.5 g of lamivudine. He presented 24 hours later to Homabay County Referral Hospital, with unresponsiveness, inability to feed and absence of playfulness. Physical examination revealed a sick-looking, ‘unconscious’ child, responding only to voice, with tachycardia, hypertension and moderate dehydration.

**Management and outcome:**

He was managed conservatively with rehydration, namely intravenous 1125 mL of 5% dextrose in 0.9% saline, and the monitoring of his neurologic status, urine output and all vital signs. He regained normal neurological function after 24 hours, and recovered uneventfully, but was lost to follow-up.

**Conclusion:**

In an area endemic for HIV and where antiretroviral drug use is commonplace, there is a need for health education to ensure that parents keep drugs out of the reach of children. In the case of a suspected overdose, parents need to be reminded to seek medical attention immediately. Physician awareness of the clinical presentation, management and challenges with an antiretroviral drug overdose is also important.

## Introduction

Antiretroviral therapy has changed infection with HIV from a fatal illness to one that is manageable.^1^ According to the UNAIDS report of 2019, 61% of new HIV infections are in sub-Saharan Africa where the incidence in Kenya is 1.02 per 1000 persons, and the overall prevalence is 4.7%. Antiretroviral drugs are used by 91% of pregnant Kenyan women living with HIV, 61% of infected children are on treatment and the number of AIDS-related deaths is now declining.^2^

Despite the widespread use of antiretroviral drugs (ARVs), paediatric overdose-related toxicity remains unreported in resource-poor settings. In 2018, Van Dam et al. described an adult parasuicide with dolutegravir/abacavir/lamivudine that resulted in lactic acidosis and hypokalemia. This was managed with intravenous fluids, potassium supplementation, the monitoring of drug levels and blood chemistries.^3^ A second adult parasuicide-overdose with dolutegravir/tenofovir/emtricitabine has also been reported.^4^ In this report the patient had a minor airway obstruction and required monitoring in an intensive care unit.

Abacavir and lamivudine are ARVs that are well absorbed orally.^5^ Abacavir is metabolised in the liver, but lamivudine is excreted unchanged in urine. Nausea, vomiting, diarrhoea, cough, fever and fatigue are side effects of both drugs.^5,6^

We report a case of suspected abacavir/lamivudine toxicity managed conservatively in a resource-poor setting. We highlight the scarcity of information and the challenges faced.

## Case report

A 3-year-old male from rural Kenya, weighing 12.5 kg, was admitted to the paediatric ward of the Homabay County Referral Hospital. This was 24 hours after ingesting four and a half bottles of abacavir 600 mg/lamivudine 300 mg combination pills prescribed for his stepbrother. Each bottle contained 60 tablets, that is, a total of 250 tablets or 15 g of abacavir and 7.5 g of lamivudine. He had been playing unattended when he ingested the tablets and afterwards appeared well and as playful as usual. He did not have a fever, rash, diarrhoea, vomiting or difficulty with breathing that would have suggested an abacavir hypersensitivity reaction. There was no report of confusion, drowsiness or seizures. He ate and slept well but the next morning was drowsy, unable to walk and feed.

He arrived looking sick, lethargic and responding only to voice commands. His airway was intact and breathing and circulation were normal. There was no pallor, jaundice, cyanosis or oedema. The eyes were sunken and the skin turgor was reduced suggesting moderate dehydration. He had a tachycardia of 156/min, a respiratory rate of 27/min, SPaO_2_ of 99% – 100%, an elevated blood pressure of 131/78 mmHg. His temperature was 37.1°C. Physical examination was unremarkable and all systems were normal. Blood glucose and full hemogram were normal. His HIV test was negative. Serum drug levels, renal and other metabolic tests were unavailable.

The child was admitted to the acute room and re-hydrated as per the Holliday-Segar method, with 1125 mL of dextrose 5% in 0.9% sodium chloride over 24 h. Vital signs and urine output were monitored hourly. Activated charcoal and gastric lavage were omitted because of the late presentation. There was improvement within 24 h. He became ambulant, fed orally and his urine output normalised. He was discharged after 24 hours of observation.

The child’s mother was counselled and advised on the safe handling of household poisons.The stepbrother’s prescription was refilled. She was single, uneducated and unemployed. She avoided discussions around paternity, marital status and HIV status. She provided no reliable contacts and never returned for follow-up.

### Ethical consideration

This article followed all ethical standards for research without direct contact with human or animal subjects.

## Discussion

Abacavir and lamivudine are commonly used in treating children with HIV. They are well absorbed orally with short half-lives of about 2 hours. While abacavir is metabolised in the liver, lamivudine is excreted unchanged in the urine. Nausea, vomiting, diarrhoea, cough, fever and fatigue are side effects of both drugs.^5,6^ They cause fatigue, and this patient had lethargy with altered consciousness. Hypersensitivity to abacavir was absent.^7^

Therapeutic daily paediatric doses of abacavir and lamivudine are 16 mg/kg and 10 mg/kg, not exceeding 600 mg and 300 mg per day.^7^ Individual doses are weight-adjusted at clinics and dispensed incrementally to reduce toxicity. The doses taken in this case far exceeded the recommended upper limit.

There is no known antidote for overdose. Management is supportive, and ideally the patient should be admitted to a high dependency unit for continuous monitoring of urine output, neurologic status and vital signs. Liver and renal function tests, with abacavir/lamivudine levels would immediately be useful to assess excretion.^8,9^ Lamivudine is dialysable,^10^ but dialysis was unavailable. The two drugs are bases and urinary alkalinisation would fail. These resources were unavailable, but he nevertheless recovered well. The few reports of adult antiretroviral overdose recommend supportive management, including intensive care. Although applicable to children, resources are often scarce. The temporal relationship between drug ingestion and presentation with signs and symptoms suggested overdose toxicity. The scarcity of investigational resources makes it difficult to be certain this was the cause of the patient’s clinical presentation.

Fatal overdoses have reduced since the introduction of child-resistant containers.^9^ These containers should have been used. Parental education on safe storage of medications away from children can prevent such cases. Inadequate maternal education likely resulted in this child’s mother being poorly informed on the proper handling of household poisons. The mother and child were lost to follow-up making it difficult to know whether the education provided to the mother had impacted on safe practices in the home.

## Conclusion

There is limited awareness of ARV overdose toxicity in children, its social factors, and management options in resource-poor settings. Antiretroviral drugs are supplied orally for prolonged periods, reducing mortality and HIV transmission. The potential for accidental overdosing remains and parental education must include the safe storage and handling of ARVs in the home.
